# Protein-Mediated Transformations of Superparamagnetic Nanoparticles Evidenced by Single-Particle Inductively Coupled Plasma Tandem Mass Spectrometry: A Disaggregation Phenomenon

**DOI:** 10.3390/ijms23031088

**Published:** 2022-01-19

**Authors:** Jacek Sikorski, Magdalena Matczuk, Agnieszka Kamińska, Joanna Kruszewska, Maciej Trzaskowski, Andrei R. Timerbaev, Maciej Jarosz

**Affiliations:** 1 Chair of Analytical Chemistry, Faculty of Chemistry, Warsaw University of Technology, Noakowskiego St. 3, 00-664 Warsaw, Poland; jsikorski@ch.pw.edu.pl (J.S.); agnieszka.kaminska.stud@pw.edu.pl (A.K.); kruszewska.j1@gmail.com (J.K.); mj@ch.pw.edu.pl (M.J.); 2 Centre for Advanced Materials and Technologies CEZAMAT PW, Warsaw University of Technology, Poleczki St. 19, 02-822 Warsaw, Poland; m.trzaskowski@cezamat.eu; 3 Vernadsky Institute of Geochemistry and Analytical Chemistry, Kosygin St. 19, 119991 Moscow, Russia; andrei.timerbaev@univie.ac.at

**Keywords:** superparamagnetic iron oxide nanoparticles, human serum, single-particle inductively coupled plasma tandem mass spectrometry

## Abstract

Progress toward translating superparamagnetic iron oxide nanoparticles (SPIONs) with specific diagnostic and therapeutic properties for clinical applications depends on developing and implementing appropriate methodologies that would allow in-depth characterizations of their behavior in a real biological environment. Herein, we report a versatile approach for studying interactions between SPIONs and proteins using single-particle inductively coupled plasma tandem mass spectrometry. By monitoring the changes in the size distribution upon exposure to human serum, the formation of stable protein corona is revealed, accompanied by particle disaggregation.

## 1. Introduction

As a collection of related diseases caused by the ability of cells to grow and proliferate uncontrollably, cancer is arguably the biggest challenge for public health care. A potential solution to this challenge may lie in nanomedicine, including the use of various nanomaterials for the diagnosis and treatment of cancer [1]. Particularly, superparamagnetic iron oxide nanoparticles (SPIONs) are widely tested as tools for drug delivery, magnetic hyperthermia, magnetic resonance imaging, catching tumor cells, etc. [2,3]. The most significant attribute of SPIONs for biomedical use is biocompatibility and biodegradability, which allows them to be trafficked via the iron metabolism pathway [4]. The characterization of the physicochemical properties of SPIONs is of the utmost importance for safe biomedical use in vivo. In particular, the colloidal stability of SPIONs in different biological media, primarily in blood serum, should be tested before introduction into a living organism [5]. However, there is a lack of research addressing this critical issue, and interactions within human serum are rarely studied in depth due to their complexity [6]. Therefore, implementing straightforward methods for a fast and reliable elucidation of the SPION behavior in complex proteinaceous media would significantly contribute to their preclinical development.

Liquid chromatography–tandem mass spectrometry (LC-MS/MS) is a powerful tool in proteomic research, which finds application for profiling the protein corona of magnetic nanoparticles [6,7]. However, the method requires laborious and time-consuming sample preparation and, because of this shortcoming, is unsuitable for high-throughput screening purposes. Among alternative techniques capable of meeting this challenge, inductively coupled plasma mass spectrometry (ICP-MS), operating in different high-resolution modes, holds great promise. This is due to the method’s proven ability of highly sensitive and interference-free monitoring of iron (constituting different nano-sized structures) in various biological environments [8,9,10,11]. Nonetheless, the reported ICP-MS-based trials overlook one crucial item, viz., whether (or not) the formation of the protein corona can prevent the agglomeration of nanoparticles. There are some indications in the literature that a steric stabilization by individual proteins, e.g., albumin [12], may lead to disaggregation. However, the techniques in use, circular dichroism spectroscopy and dynamic light scattering, are intrinsically incompatible with complex protein mixtures such as serum.

To shed light on a possible stabilization effect of serum proteins and unveil the alterations of SPIONs in human serum in toto, we propose to utilize single-particle (SP) ICP-MS/MS. Due to using collision/reaction cell technology, this technique ensures virtually no spectral interferences when detecting Fe [13]. Furthermore, compared to other high-resolution ICP-MS techniques, SP-ICP-MS/MS is much more tolerable to matrix effects as the sample is significantly diluted prior to analysis. The reason for this is the very nature of SP-based measurements, in which only a single particle is to be introduced into the detector during the dwell time. Additionally, importantly, no cumbersome sample preparation is required in contrast to, e.g., electron microscopy-based techniques [14]. However, the type and flow rate of collision/reaction gas should be carefully chosen as these operational parameters significantly influence the limit of size detection (LOSD) [15]. So far, SP-ICP-MS/MS has seldom been used to characterize SPIONs [14,15], and the effects of biological media on their stability are not yet investigated. To fill this gap, our study is focused on developing an effective assay for probing the interactions of SPIONs with serum proteins.

## 2. Results and Discussion

**Figure 1:**
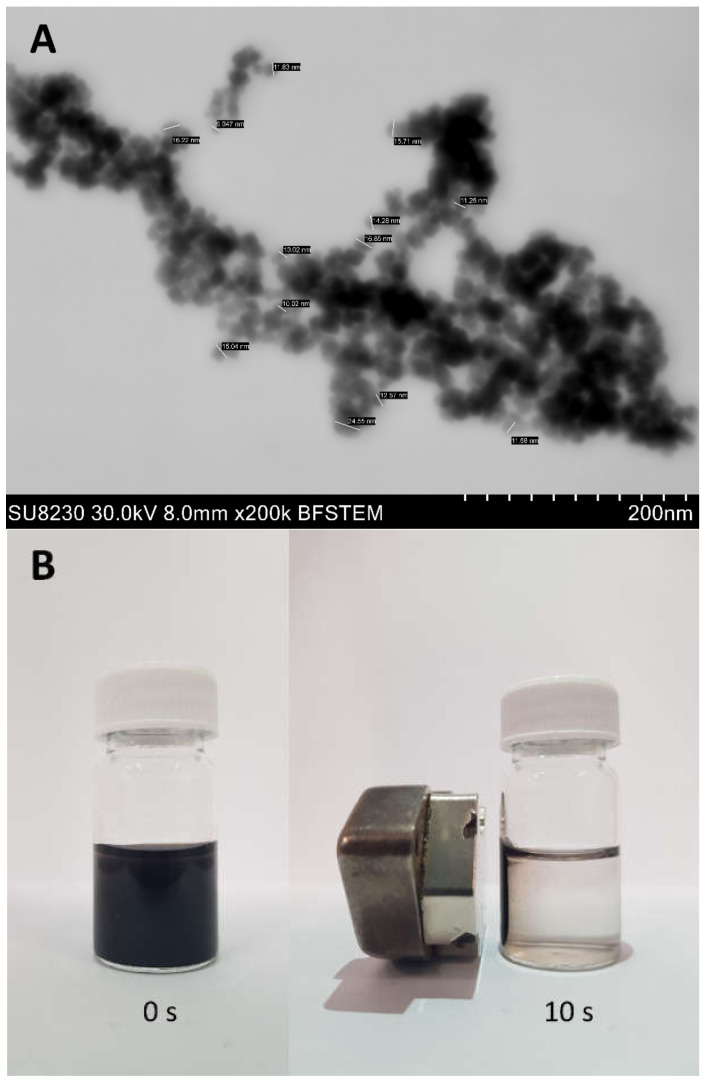
BF-STEM image (**A**) and magnetic properties (**B**) of synthesized nanoparticles.

SPIONs, synthesized as described below, were first imaged by high-resolution bright-field emission scanning transmission electron microscopy (BF-STEM; Figure 1A).

With a mean size of 16 ± 8 nm, the iron oxide nanoparticles are known to exhibit superparamagnetism [16]. The superparamagnetic properties were confirmed by subjecting the colloidal solution of SPIONs to the action of an external magnetic field, causing the particles to be instantly attracted to the magnet, as shown in Figure 1B. Any advanced surface modification that might prevent the aggregation phenomenon or impair the magnetic properties was avoided here. This is because our primary aim was to demonstrate the potential of SP-ICP-MS/MS to explore the protein-mediated stabilization of the nanomaterial.

The next experimental step was to optimize SP-ICP-MS/MS measurement conditions properly. The operational parameters, such as the flow rate of the reaction/collision gas and the width of the plasma torch injector with a dedicated nebulizer, were adjusted to obtain the lowest LOSD. For the same purpose, hydrogen was chosen as the reaction gas. Different collision gas flow rates were tested to acquire the highest possible signal-to-noise ratios of the iron (^56^Fe^+^, analyte) and gold (^197^Au^+^, from a nanoparticle standard used to calculate the nebulization efficiency) isotopes and to minimize spectral interferences. Then, the torches with different injector widths were compared (1.5 vs. 2.5 mm) with the LOSD criterion in mind. The application of the torch with a narrower width turned out to generate better results. The optimized operational conditions, shown in Table 1, guaranteed the good quality of the size characterization of SPIONs.

**Figure 2:**
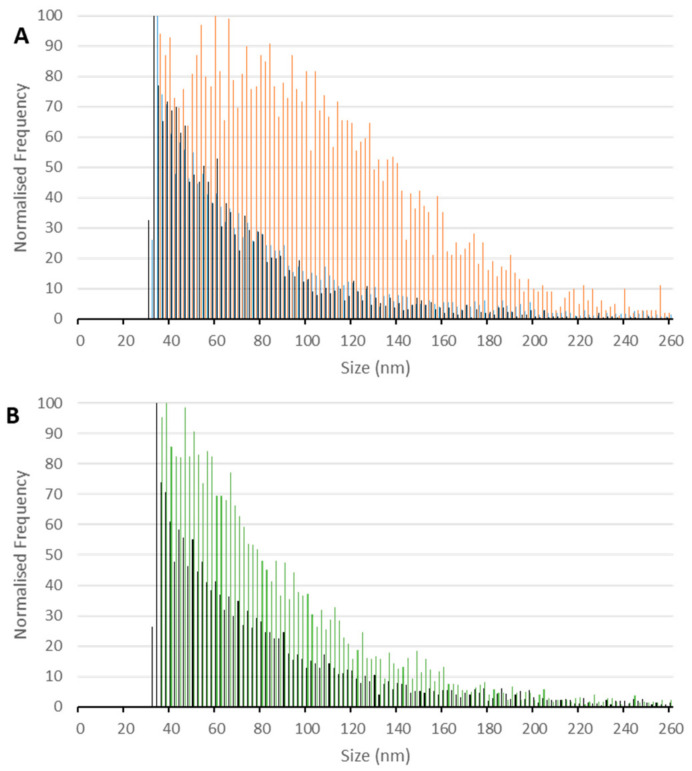
Size distribution of original SPIONs ((**A**), orange), incubated with serum without ((**A**,**B**); black) and with ((**A**), blue) ultrafiltration, and incubated with albumin ((**B**), green), without ultrafiltration. The concentration of SPIONs in all suspensions—500 ng/L Fe.

Using the optimized assay, a detailed study of the SPIONs under investigation was performed, focusing on the effect of human serum medium on the nanostructural stability. A 500 ng/L Fe concentration was kept in all measured samples, requiring significant dilution factors prior to SP-ICP-MS/MS analysis. Shown in Figure 2A (orange color) is the size histogram of initial SPIONs. A wide distribution of core diameters confirms the particle agglomeration, which is in accord with the BF-STEM testing (Figure 1A). To prove that the protein corona prevents the particles from touching each other [17], the standard suspension of SPIONs was added to the human serum diluted by 10 times with 10 mM phosphate buffer (pH 7.4) containing 100 mM NaCl. The mixture was incubated at 37 °C for 1 h, this incubation time being sufficient for protein adsorption to reach an equilibrium state [18]. The SP-ICP-MS/MS assaying data imply a drastic change in the structural organization of SPIONs in the serum environment (cf. orange and black patterns in Figure 2A), resulting in reduced agglomeration. However, a question remains unanswered: how stable are the resultant protein nanoconjugates? To gain an insight into this, we repeated the measurements after the ultrafiltration of the incubated sample. As can be judged from the comparison of the blue and black traces in Figure 2A, removing excessive, unattached proteins from the mixture causes no notable changes in the size profile. No re-agglomeration of the nanomaterial is evident, and the corona seems to retain stability after separating the surplus of proteins. Additionally, it is noteworthy that similar signal intensities were recorded before and after ultrafiltration.

Finally, the effect of albumin on the agglomeration of SPIONs was examined to conceive the corona composition. Even though this is the most abundant serum protein, other proteins, e.g., apolipoproteins or immunoglobulins [19], may significantly contribute to building the surface biomolecular layer(s). The results presented in Figure 2B indicate a reduction in agglomeration but not so massive as that which occurs in the whole serum milieu. This finding proves that proteins other from albumin can display an impact on the effective organization of particles in a biofluid by the mechanism of corona formation.

## 3. Materials and Methods

### 3.1. Chemicals

Iron(II) sulfate heptahydrate, iron(III) sulfate hydrate, sodium dihydrogen phosphate, sodium hydrogen phosphate, sodium chloride, and sodium hydroxide were purchased from Sigma-Aldrich (St. Louis, MO, USA). Nitric acid was the product of Avantor (Radnor, PA, USA). Single-element gold and iron ICP standard solutions were obtained from Merck Millipore (Darmstadt, Germany). Gold nanoparticle suspension (50 nm) was purchased from British Biocell International (Cardiff, UK). Human serum and albumin from human serum were purchased from Sigma-Aldrich (St. Louis, MO, USA). The ultrapure water used throughout this study was obtained from Millipore Elix 3 system (Merck Millipore, Molsheim, France).

### 3.2. Synthesis of SPIONs

SPIONs were synthesized via the coprecipitation of Fe^2+^ (3 mL of 0.05 mol/L FeSO_4_) and Fe^3+^ (1 mL of 0.05 mol/L Fe_2_(SO_4_)_3_) salts. The formation of nanostructures was achieved by adding 4 mL water at 80 °C, and 2 mol/L NaOH (2 mL) was used as a precipitating agent. After 5 min of stirring at 300 rcf, the obtained nanomaterial was magnetically separated from the reaction mixture and then rinsed with 0.1 mol/L nitric acid (1 mL) and several times with water. Finally, the SPIONs were suspended in water.

### 3.3. Characterization of SPIONs

A Hitachi Su8230 microscope at an accelerating voltage of 30 kV was used to obtain BF-STEM images for size characterization. Samples were imaged using gold TEM grids coated with Lacey carbon film. The grid was immersed in samples in the form of water suspensions and dried before observation.

### 3.4. SPIONs-Proteins Sample Preparation

Incubation buffer (10 mM phosphate buffer containing 100 mM NaCl, pH 7.4) was prepared using Na_2_HPO_4_, NaH_2_PO_4_, and NaCl. For investigation of nanoparticle–protein interactions, the synthesized SPIONs were incubated with human serum or albumin, diluted by 10 times with phosphate-buffered saline, for 1 h at 37 °C. After incubation with human serum, the mixture was subjected to ultrafiltration through 10 kDa filters (Amicon Ultra-0.5 mL) at 10,000 rcf for 30 min.

## 4. Conclusions

The potential of SP-ICP-MS/MS as a viable tool for the SPION–protein analysis has been verified in this proof-of-principle study. The proposed method allows for a fast and straightforward monitoring of the changes in the structure of SPIONs upon interaction with serum proteins. We believe that it might find a niche in examining other nanoparticle-protein combinations and thereby help shortlist the investigational nanomaterials before in-depth preclinical testing.

## Figures and Tables

**Table 1:** Optimal SP-ICP-MS/MS parameters.

Parameter	Setting
Sample depth	8.0 mm
Torch width	1.5 mm
Nebulizer gas (Ar) flow	0.95 L/min
Reaction gas (H_2_) flow	5.00 mL/min
Sampler and skimmer cones	Pt
